# Visual Threat Location Impacts Brain‐Wide Visual Adaptation Networks

**DOI:** 10.1002/cne.70182

**Published:** 2026-07-14

**Authors:** Tessa Mancienne, Emmanuel Marquez‐Legorreta, Marielle Piber, Maya Wilde, Gilles Vanwalleghem, Itia Favre‐Bulle, Ethan K. Scott

**Affiliations:** ^1^ Department of Anatomy and Physiology University of Melbourne Melbourne Victoria Australia; ^2^ Section Cell Biology, Neurobiology and Biophysics, Department of Biology University of Utrecht Utrecht the Netherlands; ^3^ Institut Pasteur Université Paris Cité Paris France; ^4^ Department of Cybernetics Faculty of Electrical Engineering Czech Technical University Prague Czechia; ^5^ Department of Molecular Biology and Genetics Aarhus University Aarhus Denmark; ^6^ Queensland Brain Institute University of Queensland St. Lucia Queensland Australia; ^7^ School of Mathematics and Physics University of Queensland St. Lucia Queensland Australia

**Keywords:** calcium imaging, escape, habituation, neural adaptation, tectum, vision, zebrafish

## Abstract

Habituation is a simple form of nonassociative learning that is characterized by a decrease in response to a repetitive stimulus. As escape responses can be energetically costly and disruptive to normal behavior, it is important that prospective prey learn whether a perceived stimulus is a genuine threat or an innocuous stimulus that they can ignore. In response to a visual looming stimulus, larval zebrafish perform a characteristic escape swim that reliably habituates, and because they are small and transparent, they have been an important model for characterizing brain‐wide activity patterns during habituation. In this study, we explore the spatial properties of visual adaptation to gauge whether it is mediated by local, regional, or brain‐wide circuits. We present repetitive visual loom stimuli either in a fixed position in visual space or in variable positions, while also performing brain‐wide calcium imaging. Across the brain, we identify both neural responses that are specific to looms at particular positions within the visual field and responses that occur regardless of where the loom is presented. By quantifying the degree of adaptation across these responses, we show that brain‐wide adaptation occurs more rapidly when the position of the loom remains unchanged and that alternate looms occurring in different parts of the visual field minimally contribute to adaptation for looms at the original position. We found that the tectum, homologous to the superior colliculus, has response profiles and spatial sensitivity indicative of important contributions to this position‐specific visual adaptation.

## Graphical abstract

Mancienne et al. explored the impacts of varying stimulus position on visual adaptation in zebrafish larvae. They found that responses to a looming stimulus in a particular visual position adapt quickly, but that looms elsewhere in the visual field have little impact on adaptation to the reference stimulus. Brain‐wide calcium imaging reveals the brain structures in which this adaptation occurs and the ways in which signals from different visual positions interact during visual adaptation.

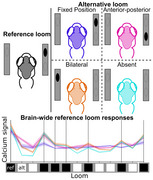

## 1 Introduction

Across the animal kingdom, predator evasion is critical for survival and involves performing appropriate behaviors in response to threatening sensory stimuli (Card and Dickinson 2008; Marquez‐Legorreta et al. 2020; Yilmaz and Meister 2013). Although multiple sensory modalities can elicit escape responses (Burgess and Granato 2007; Eaton et al. 1977; Li et al. 2021; Maguire et al. 2011; McHenry et al. 2009; Ohyama et al. 2013; Poulsen et al. 2021; Privat et al. 2019; Y. Wang et al. 2023), vision provides particularly rich information on predators' position, movement, and speed (Burgess and Granato 2007; Dunn et al. 2016; Eaton et al. 1977; Holmqvist and Srinivasan 1991; Marquez‐Legorreta et al. 2020; McHenry et al. 2009; Shang et al. 2018; Temizer et al. 2015; Yilmaz and Meister 2013). These characteristics help potential prey determine the saliency of a threat and shape their responses accordingly. However, unless a genuine threat exists, escape responses can become unnecessarily disruptive to normal behavior and energetically costly. As a result, complex circuitry has evolved to distinguish genuine threats from similar stimuli that are innocuous (Lenzi et al. 2022; Marquez‐Legorreta et al. 2020).

An ethologically important example is habituation. Habituation is a simple form of nonassociative learning that is highly conserved across the animal kingdom (Friedman 1972; Glanzman 2009; Laming and McKinney 1990; Miller et al. 2022). This learning is characterized by a gradual decrease in the probability of a behavioral reaction in response to a repetitive innocuous stimulus and can be distinguished from sensory adaptation or motor fatigue by its sensitivity to different stimulus properties, its recovery after a time of stimulus absence, and the animal's ongoing responsiveness to different stimuli (Rankin et al. 2009). In larval zebrafish, visual habituation is often studied using repeated presentations of a looming stimulus—termed a “loom”—an expanding 2D shape that mimics an approaching predator (Bhattacharyya et al. 2017; Dunn et al. 2016; Fotowat and Engert 2023; Marquez‐Legorreta et al. 2022; Temizer et al. 2015). The resulting behavior is a well‐characterized suite of avoidance responses that habituate over the course of repeated loom presentations (Eaton et al. 1977; Fotowat and Engert 2023; Marquez‐Legorreta et al. 2022). The broad decrease in neural activity observed during habituation is often referred to as neural adaptation and may be a result of different cellular or molecular mechanisms occurring at specific sites along the sensory pathway, such as a decrease in presynaptic quanta release, increased inhibitory drive, or changes in postsynaptic excitability (Burrell et al. 2001; Castellucci and Kandel 1974; Das et al. 2011).

The tectum, homologous to the mammalian superior colliculus, is a key center for spatial and temporal integration of sensory stimuli and is proposed to play an important role in the sensorimotor gating that occurs during habituation (Dutta and Gutfreund 2014; Isa et al. 2021; Marquez‐Legorreta et al. 2022; Netser et al. 2010). In addition, the tectum is known to encode and extract different features of visual stimuli, such as size and position, to drive appropriate responses; a smaller stimulus in the anterior visual field is likely to elicit predatory behavior, while a larger stimulus in the posterior visual field is likely to elicit an avoidance response (Bianco et al. 2011; Bianco and Engert 2015; Förster et al. 2020; Helmbrecht et al. 2018). To dissect the neural circuits mediating visual habituation, a loom can be broken down into two components: a decrease in luminance and the perceived motion of expanding edges (Fotowat and Engert 2023; Heap et al. 2018; Mancienne et al. 2021). Each of these properties is shown to modulate aspects of escape behavior: a dim‐sensitive retino‐thalamo‐tectal circuit determines both the probability of an escape and the direction in which it is elicited, while edge‐ and motion‐detecting retino‐tectal circuits influence the probability of an escape (Heap et al. 2018). Dim‐sensitive and edge detection neurons, as well as neurons encoding the size and position of a stimulus, can be found across the tectum, suggesting a role for the tectum in the integration of the different sensory components of looms. These features position the tectum as a likely site for adaptation (Heap et al. 2018; Mancienne et al. 2021; Robles et al. 2014; K. Wang et al. 2020).

The details of how activity changes across the visual pathway as adaptation and habituation proceed are still not fully understood, and this prompts our current exploration of whether adaptation is mediated by local tectal or global brain‐wide circuits. In this study, we probed the spatial properties of these circuits by conducting light‐sheet calcium imaging on stationary larval zebrafish while delivering looms to various positions within the visual field, assessing how the presence of these differing stimuli influences adaptation across the brain. This approach has allowed us to identify neural responses that are specific to the location of looms, as well as responses that are location independent, and has shown which neurons across the brain have reduced responses during network‐wide adaptation. By characterizing the anatomical distributions of these responses across the brain and further quantifying the degree of brain‐wide adaptation, we show that this sensorimotor gating is likely mediated by local circuits, whereby looms in a specific position within the visual field drive adaptation without appreciable input from looms presented elsewhere within the visual field.

## 2 Results

### 2.1 Imaging Positional Loom Responses Across the Zebrafish Brain

**FIGURE 1:**
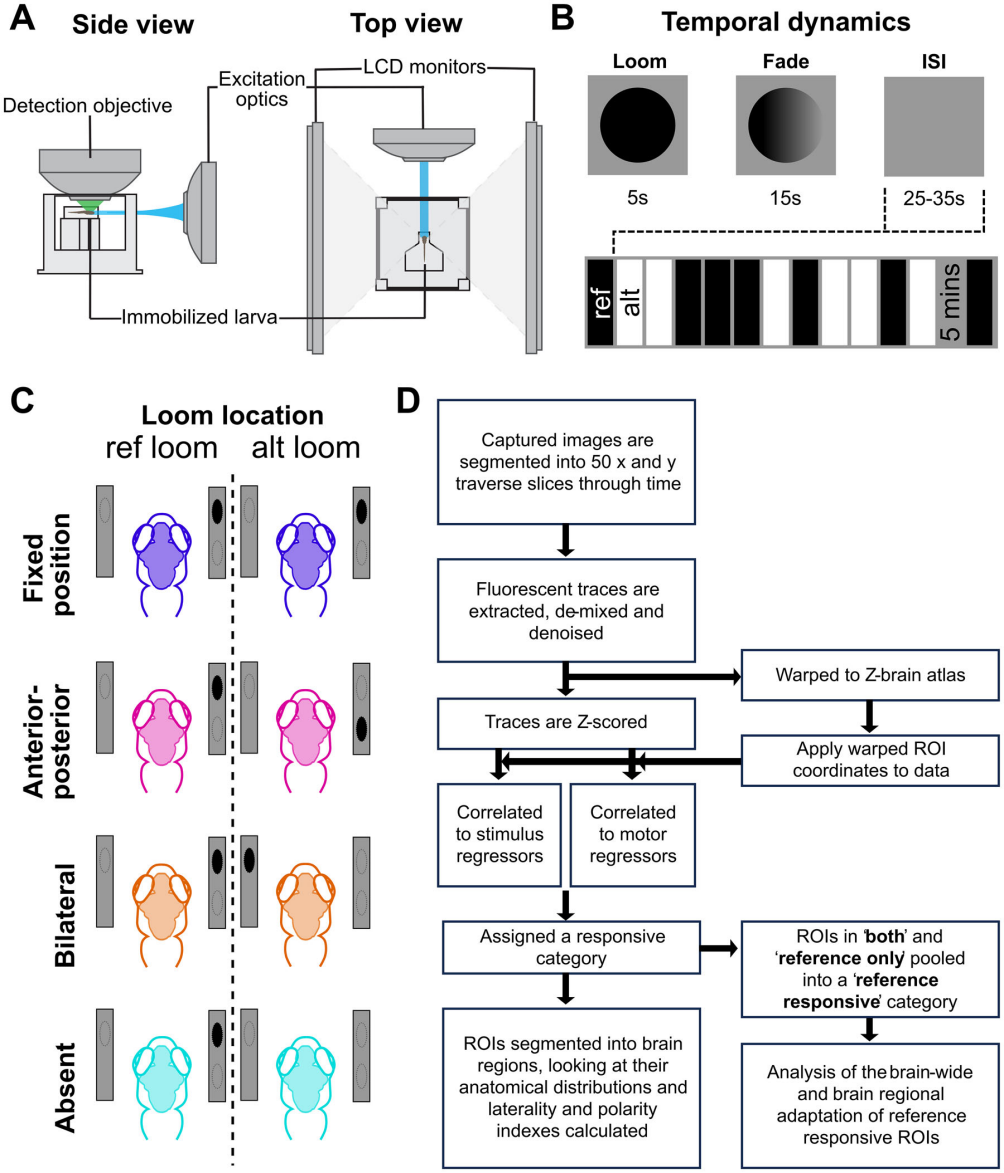
Experimental paradigm. (A) Schematic of light‐sheet preparation used for calcium imaging. (B) Temporal properties of looming stimulus used, where looms expanded exponentially over 5 s and faded linearly for 15 s. The interstimulus intervals for the train were 25–35 s, and Loom 13 was presented after a 5‐min recovery period. (C) Spatial properties of the looming stimuli used, showing where reference looms and alternate looms were positioned in visual space relative to the embedded fish. (D) Summary of the analysis pipeline used to process calcium imaging data.

We performed brain‐wide, cellular‐resolution calcium imaging in 6‐day postfertilization (dpf) zebrafish (*Danio rerio*) larvae using a house‐built light‐sheet microscope to observe the calcium indicator GCaMP6s in the nuclei of all neurons (Figure 1A). The resulting volumetric imaging data were analyzed using CaImAn to identify regions of interest (ROIs) generally corresponding to individual neurons (see Section 4). Across 35 animals that were imaged in this manner, we segmented 19,161 ± 6153 ROIs (mean ± *SD*) per animal and extracted activity profiles for each ROI (at a volumetric imaging rate of 2 Hz) for the duration of the experiment.

To explore the networks underlying visual adaptation, we employed four stimulus trains comprising visual looms in varying positions (Figure 1C). Within each train, some of the looms (referred to as reference looms) were delivered in the right anterior eye field, while the other looms (alternate looms) were delivered to different locations in visual space. The temporal properties and order of reference and alternate looms were kept consistent across the different stimulus trains so that reference and alternate responses could be compared across groups (Figure 1B). The first train, fixed position, had alternate looms in the front right field, matching the reference looms. This consistent positioning of the looms was intended to drive maximal adaptation across the brain. In the anterior–posterior train, alternate looms were delivered in the posterior right eye field, a preparation intended to reveal contributions to adaptation from looms in a different part of the same eye field. The bilateral train had alternate looms in the anterior left eye field, probing for contributions by looms from the opposite eye. Finally, the absent train served as a negative control, with no loom occurring during alternate trials (Figure 1C).

### 2.2 Loom Position Impacts the Number, Positions, and Response Profiles of Loom‐Responsive ROIs

**FIGURE 2:**
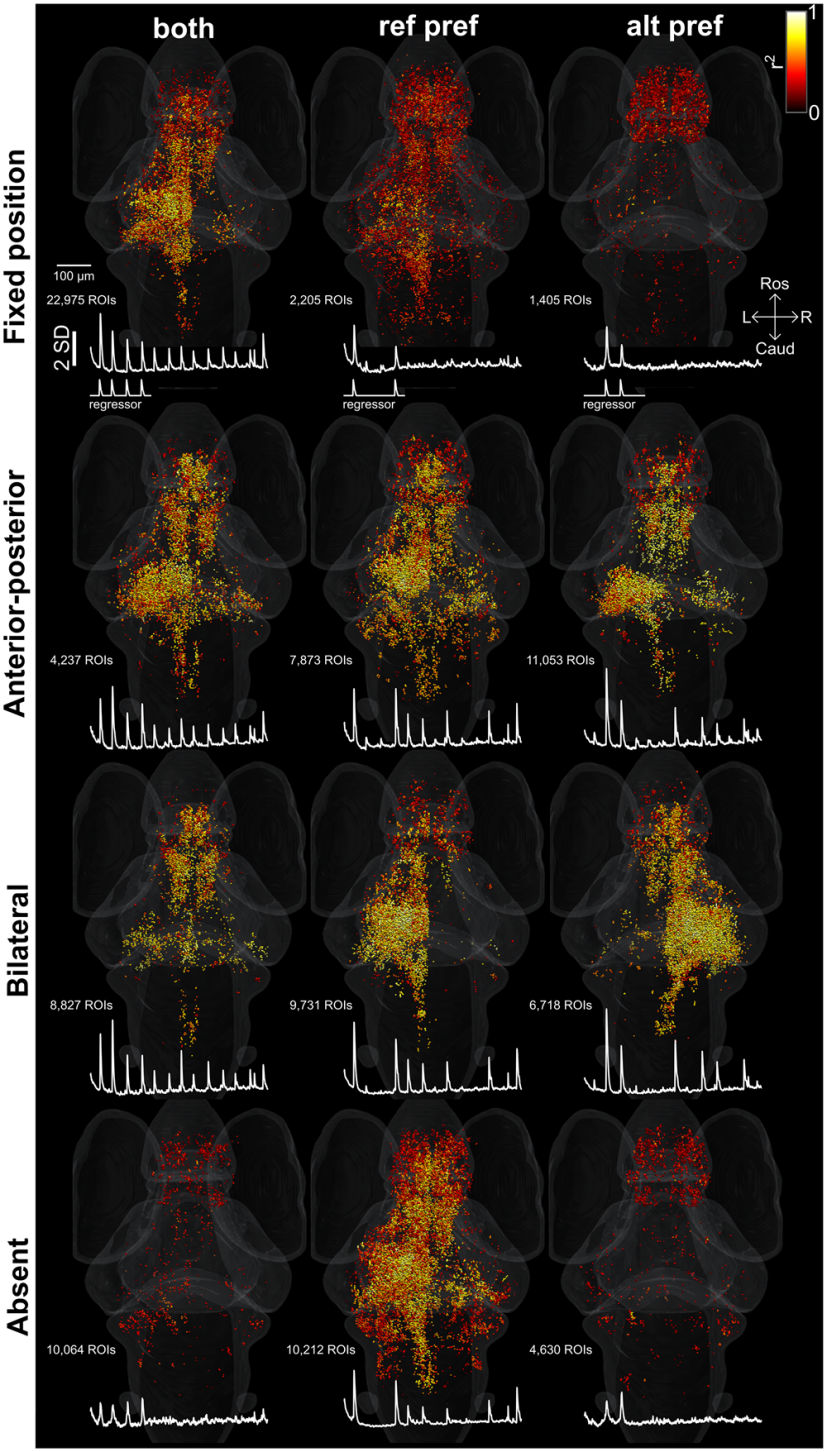
Brain‐wide responses to visual stimuli. Distributions of visually responsive ROIs pooled together from each of the four stimulus trains (fixed position, *n* = 8; anterior–posterior, *n* = 9; bilateral, *n* = 8; absent, *n* = 10). This analysis yielded three categories: “both” responses, “reference preferring” responses and “alternate preferring” responses. Mean calcium traces for all ROIs within that category are shown below the brain distribution plots, along with the regressors used to pull those ROIs.

Using stimulus regressors built by placing a simulated calcium spike at the time points of the first four looms (Figure 2, top), we separated visually responsive ROIs into three categories: (1) “reference preferring”: those responding principally to reference looms, (2) “alternate preferring”: those responding principally to alternate looms, and (3) “both”: those showing similar responses to both types of stimuli (Figure 2). Because of the steep adaptation that occurred in many ROIs, we based this categorization on responses to the first four stimuli (1: reference, 2: alternate, 3: alternate, and 4: reference; Figure 2). Next, we used motion correction performed during preprocessing and built motion regressors to filter out motor responses to the stimuli. We carried out an exploration of *r* ^2^ thresholds for both the motor and stimulus regressors to find values that give a good balance between filtering out motor activity and maintaining interesting sensory information (see Section 4).

Figure 2 shows that the distribution of “both” ROIs in the anterior–posterior train was similar to those in the fixed position train, but there were more ROIs in the “reference preferring” and “alternate preferring” categories. This appears to be due to the different positions of the reference and alternate looms in the left eye field; retinotopic projections from the eye, especially to the tectum, deliver these signals to distinct areas of the tectum (Burrill and Easter 1994; Nikolaou et al. 2012; Robles et al. 2014). This interpretation is supported by the concentration of “alternate responsive” ROIs in more caudal positions of the tectum compared to the “reference responsive” ROIs. The large number of “both” ROIs in this condition suggests that many neurons respond regardless of the loom position or are responsive to a large enough region of visual space to capture both stimuli.

Also reflecting the nature of the stimuli, the bilateral train resulted mostly in “reference preferring” and “alternate preferring” ROIs. These were strongly lateralized, consistent with the fact that all retinal projections are contralateral in zebrafish (Robles et al. 2014; Stuermer 1988). A smaller number of “both” ROIs were observed, likely representing neurons responsive to looms in any part of the visual space.

Finally, as expected, the absent train was dominated by “reference preferring” ROIs, since the absence of alternate stimuli prevented responses in the “alternate preferring” and “both” categories.

These data demonstrate that loom responses are widespread, that they conform to predictions based on known anatomical connections, and that different ROIs have different levels of spatial selectivity. We next looked in more detail at responses within several brain regions to gauge the degree to which their ROIs are responsive to looms in general or to looms in particular positions.

### 2.3 Different Brain Regions Respond Differently to Positional Loom Stimuli

**FIGURE 3:**
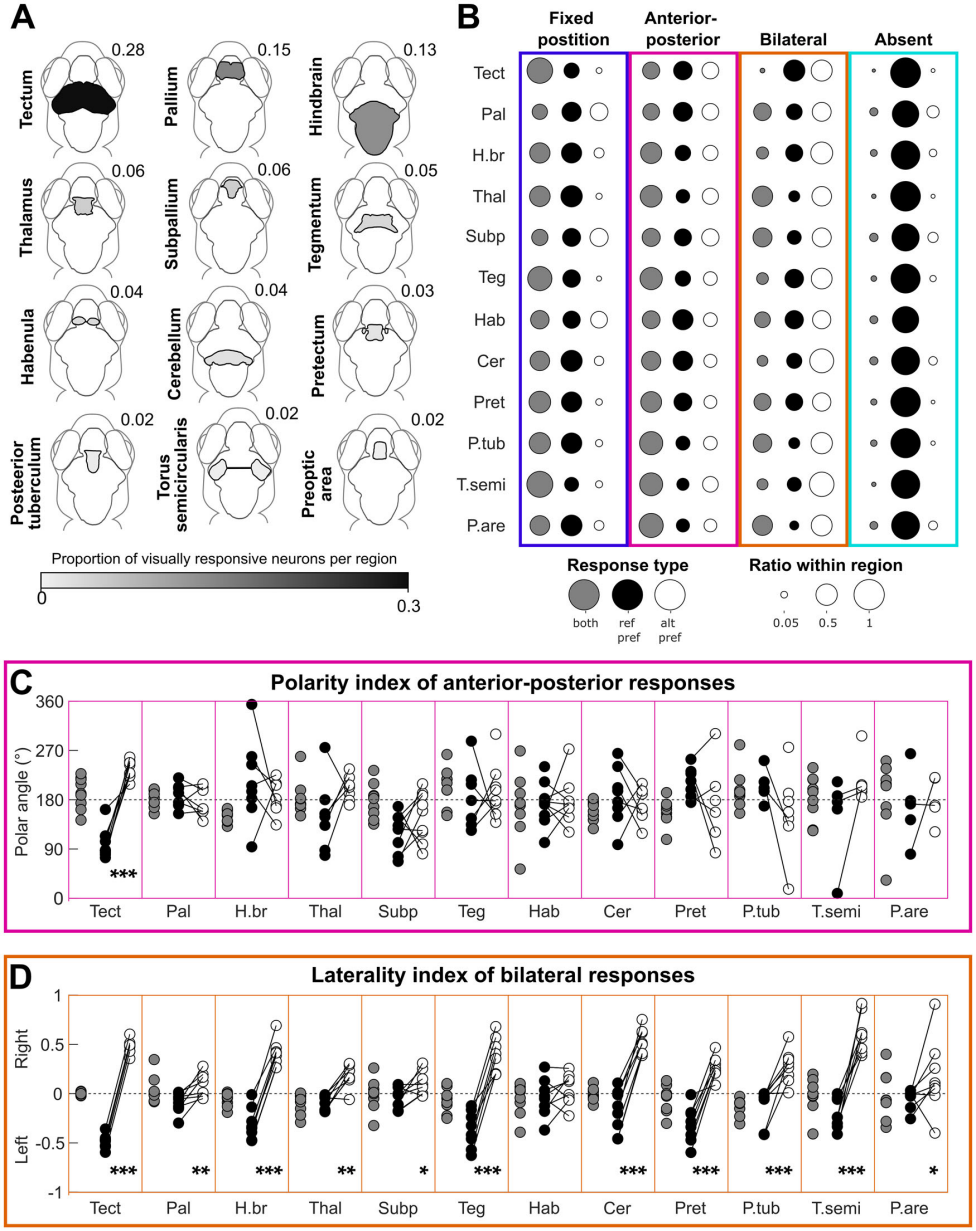
Regional properties of brain‐wide visual loom responses. (A) The proportion of visually responsive ROIs within each brain region. (B) The proportion of response type within each brain region for each of the stimulus trains. (C) The polarity index of brain regions within the anterior–posterior train. (D) The laterality index of brain regions within the bilateral train. For (C) and (D), significance was tested using the Mann‒Whitney *U* test (*p < 0.05, **p < 0.01, ***p < 0.001).

To quantify the distribution of loom responses, we first pooled all response types together and calculated their average distributions across known visual processing brain regions (Figure 3A, see Methods). A large proportion (28%) of ROIs within the tectum were loom responsive, and such cells were also present in other retinorecipient regions, such as the thalamus (6%) and pretectum (3%). Responses were found in higher order regions such as the pallium (15%) and subpallium (6%), and additionally found in the hindbrain (13%), which likely correspond to premotor regions involved in escape responses.

Next, we looked at the ratio of the different response types across these brain regions. Predictably, the fixed position train resulted mostly in ROIs in the “both” category, since reference and alternate looms are the same in this condition. ROIs whose responses were predominantly to the very first stimulus appeared in the “reference preferring” category, since the first stimulus is a reference loom (Figure 3B). The absent train resulted in the majority of ROIs falling in the “reference preferring” category, as fish in this train received no stimulation during the alternate time points.

This analysis serves two purposes. First, as just described, it provides sanity checks for our stimulus presentation, calcium imaging, and analysis pipeline. The fulfillment of firmly grounded expectations (mostly “both” responses in the fixed position experiments and mirror images across the tecta in the bilateral experiments, for example) suggests that this approach is sound. The second purpose is to allow us to gauge the degree of overlap in responses of ROIs to the reference and alternate looms for each stimulus train. This is based on the rationale that the neurons driving adaptation must be involved, directly or indirectly, in processing all stimuli that contribute to adaptation. The best candidates for mediating adaptation, therefore, will be neurons either in the sensory pathways for both reference and alternate looms or in a position downstream of separate sensory pathways for both stimuli.

As a means of quantifying the overlap and spatial properties of reference and alternate loom responses for each brain region, we calculated polarity and laterality indices (representing rostral‐caudal and left‐right separation, respectively, see Section 4). The polarity index gives a polar angle that represents how spatially segregated responses to the anterior and posterior visual field are within brain regions. In the anterior–posterior train, an even split of all response types within the optic tectum (Figure 3B) suggests that while there were distinct stimulus‐specific responses, there may have been some overlap within the visual field when presenting stimuli. A significant separation in the rostral‐caudal positions between reference (anterior) and alternate (posterior) responses Figure 3C) shows that these stimuli are recruiting at least partially non‐overlapping regions of the tectum. This separation, caused by the topographic map between the retina and tectum, will test the idea that brain‐wide adaptation can be driven by different stimuli as long as they recruit neighboring sensory pathways. In all other regions tested, there was no significant rostral‐caudal separation between responses to anterior and posterior stimuli (Figure 3C).

The laterality index gives a score of 0 when responses to the left and right visual field within the region are evenly distributed across the hemispheres, while scores closer to 1 or −1 occur in regions that have imbalanced responses weighted toward the right or left visual field, respectively. The bilateral train presented the most spatially separated stimuli, and as expected, this laterality is strongly represented in the tectal responses (Figure 3D). While a majority of ROIs respond specifically to stimulation by looms in either the left or right visual field, certain brain regions, namely, the pallium, subpallium, and thalamus, contain populations of ROIs that are responsive to both left and right stimuli (Figure 3B). These may be bilaterally integrated responses or those that are part of the main visual circuitry responding to the presence of a visual stimulus, regardless of the position.

### 2.4 Whole‐Brain Adaptation of Reference Responses

**FIGURE 4:**
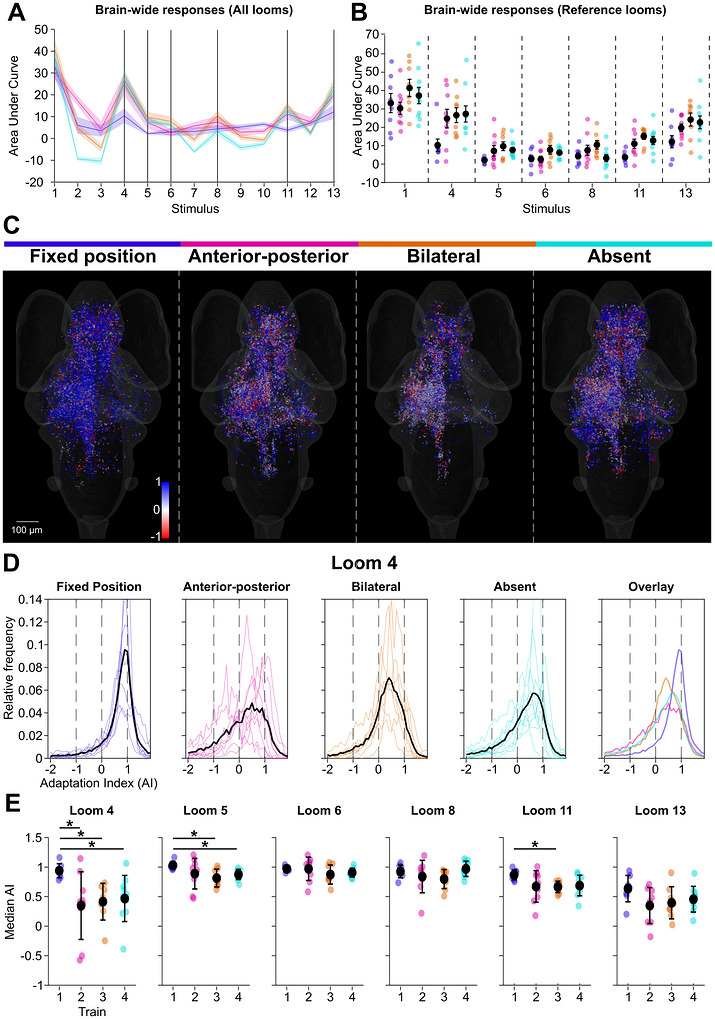
Brain‐wide adaptation of reference responsive ROIs. (A) ROIs from the categories “reference preferring” and those responding to “both” were pooled together as reference responsive. The area under the curve was calculated for responses at each stimulus presentation and averaged within fish and within trains. (B) Average AUC of reference loom responses per fish within each stimulus train. (C) Spatial distribution of responses across the brain, colored by their adaptation index (AI); the opacity of each ROI is also determined by their absolute AI value. (D) Distributions of AI of Loom 4 within each stimulus train. (E) The average median of the adaptation index distribution across the four stimulus trains across all the reference loom trials. Significance was tested using the Kruskal‒Wallis test and corrected for multiple comparisons with Bonferroni correction (*p < 0.05).

To look at adaptation across the entire brain, we pooled together “reference preferring” and “both” ROIs as “reference responsive”. In isolating these responses, we can address how off‐target stimuli influence adaptation since these stimuli are kept constant in timing and position across the four stimulus trains. Figure 4A shows that brain‐wide responses from “reference preferring” and “both” ROIs are similar for all four stimulus trains by Stimulus 6. However, at Stimulus 4, our second reference loom following the presentation of two alternate looms, there is a markedly weaker response in the “fixed position” group compared to the other three groups (Figure 4A, with reference looms shown in more detail in Figure 4B). This result suggests that the second and third looms, presented in the same visual location, contributed to greater adaptation than was seen for the “absent” group, which did not receive the second and third looms. In addition, the results hint that looms in another part of the same eye field, or on the opposite side of the visual field, contribute little to the adaptation of ROIs responding to the reference loom.

Further investigating the extent of adaptation, we calculated the adaptation index of each ROI at all reference loom time points relative to the first trial (see Section 4) and looked at the distribution across the brain, providing a granular readout of how much the brain had adapted (Figure 4C–E). Here, a value of 1 represents complete adaptation, 0 indicates similar responses across both time points, and negative values indicate increased responses relative to the first. Reflecting the averaged results above, the “fixed position” network in Trial 4 is dominated by strongly attenuated responses covering a majority of all brain regions. In contrast, ROIs that have not adapted or that have actually increased their responses are found throughout the networks for the other three stimulus trains.

Population‐wide distributions of AI (Figure 4D) show an expected shift toward greater adaptation in the “fixed position” group compared to the others. These results imply that when animals receive constant stimulation in one part of their visual field, there is a greater number of ROIs habituating more rapidly than when there are off‐target stimuli or no stimuli presented at all. When we analyze this effect at the level of the animals (Figure 4E), we find significantly stronger adaptation in the “fixed position” group than in the others and similar levels of adaptation across the other three groups. This trend persists weakly in loom 5 before disappearing in Loom 6 (Figure 4B). Loom 11, which is similar to Loom 4 in that it follows two alternate looms, shows a similar trend to Loom 4's, but to a lesser and mostly insignificant degree. Notably, there are no analyses or timepoints with significant differences in responses to reference looms across the “anterior–posterior,” “bilateral,” or “absent” stimulus trains. This result suggests that off‐target stimuli, whether they are elsewhere in the same eye field or presented to the other eye, do not contribute to adaptation of the reference loom. For larval zebrafish, and in the preparation that we present here, adaptation appears to rely wholly on visual processing circuitry that is tightly restricted in visual space.

**FIGURE 5:**
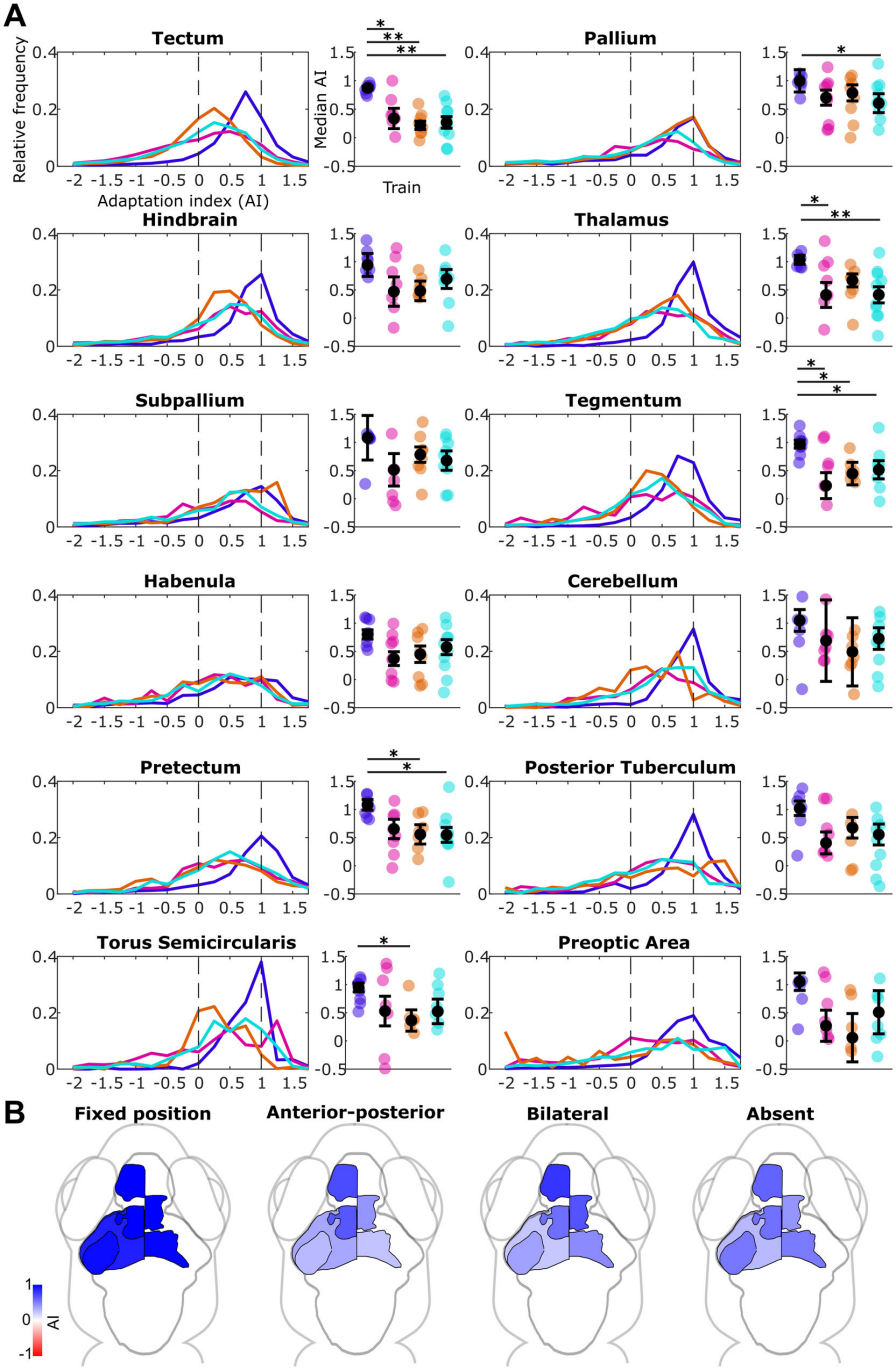
Regional adaptation of reference responsive ROIs. (A) Distributions of AI of Loom 4 within each stimulus train within each brain region, along with shows the average median of adaptation index distribution across the four stimulus trains, across all the reference loom trials. Significance was tested using the Kruskal‒Wallis test and corrected for multiple comparisons with Bonferroni correction (*p < 0.05, **p < 0.01, ***p < 0.001). (B) Schematic of brain regions that show at least one significant difference, colored by the average median adaptation index.

Next, we looked at the AI distribution of “reference preferring” ROIs within different visually responsive brain ROIs to investigate whether specific regions drive the results above. Overall, we find that most regions follow a similar trend to what we report at the whole‐brain level, where there is a greater shift toward adaptation in fish receiving constant stimulation in the “fixed position” train relative to the other trains (Figure 5A). This appears more prominent in the tectum, hindbrain, thalamus, tegmentum, cerebellum, pretectum, posterior tuberculum, torus semicircularis, and preoptic area, where the distributions for the “fixed position” train have a distinctly taller peak at an AI of 1. Notably, the “fixed position” adaptation is significantly greater in the tectum and tegmentum relative to all the other trains, the thalamus significantly greater than “anterior–posterior” and “absent,” and the pretectum significantly greater than “bilateral” and “absent” (Figure 5A,B). These regions may be involved in circuits that mediate adaptation to localized parts of the visual field.

## 3 Discussion

Avoidance responses are critical to survival but come at a cost of energy expenditure and disruption to routine behavior. As such, the decision to perform an escape response is important and must take context and previous experience into account. Habituation is the learning process by which the sensorimotor gate is tipped toward ignoring an otherwise threatening stimulus when it has repeatedly occurred without consequence (Rankin et al. 2009). In this study, we asked whether local, regional, or global networks mediate adaptation to a repetitive threatening visual stimulus. To do so, we imaged activity across the larval zebrafish brain while presenting stimulus trains consisting of a repeating loom stimulus that differed in its position in visual space. Across these trains, looms that were kept spatially consistent were termed reference looms, serving as a reference point for analyses and interpretations, while those that appeared in a different position were termed alternate looms. This allowed us to study the effect of these alternate off‐target loom presentations on the subsequent adaptation to reference looms.

As expected, when no alternate stimuli are presented (our absent train), no adaptation results from these trials, as these trials effectively provide a longer interstimulus interval between reference looms presented in the same position, a feature of the stimulus train previously shown to reduce the rate of habituation (Davis 1970; Laming and McKinney 1990; Marquez‐Legorreta et al. 2022; Post and Von Der Emde 1999; Rankin et al. 2009; R. F. Thompson and Spencer 1966). Interestingly, we find that the presentation of alternate looms to either the posterior part of an eye's visual field or to the opposite eye generates similar reference responses to those seen from the absent train, suggesting that these off‐target stimuli do not contribute to the brain‐wide adaptation (Figure 4D). This effect is strongest when the second reference loom (Loom 4) appears after two presentations of the alternate loom trial (Looms 2 and 3). As the train proceeds with more reference looms, responses adapt rapidly, reaching similar levels across the four trains. Echoes of the original effect can be seen in subsequent reference looms that are preceded by alternate looms, but these effects were generally not significant. We also identified neurons that responded regardless of where the stimulus was positioned within the visual field. These neurons may be part of previously described core loom perception circuits or otherwise located upstream of the neurons involved in sensorimotor gating (Marquez‐Legorreta et al. 2022).

We interpret these results to mean that rapid loom adaptation depends on the repetitive stimulation of the same region of visual space; neither stimuli presented to the contralateral eye nor to a different retinal position in the same eye contributes to brain‐wide adaptation to the reference stimulus. This interpretation meshes with other observations within and beyond the current study. A previous study found that switching the loom presentation between the eyes of a larval zebrafish during a habituating train generates a recovery in the escape response (Fotowat and Engert 2023), a finding that we have also made in our calcium imaging data where we have identified populations of ROIs that increase in response when the loom switches between the back and the front of the eye (anterior–posterior), between the two eyes (bilateral), and when there is a gap without a stimulus (absent). Mediating this partially recovered response, they (Fotowat and Engert) identified a group of dim‐sensitive neurons (responsive to a global dimming stimulus) that potentiate upon repeated presentation of the stimulus and dampen responses to subsequent looms (Fotowat and Engert 2023). From these results, they propose that habituation is a result of tectal circuitry consisting of potentiating dim‐sensitive neurons locally inhibiting loom‐sensitive neurons that feed onto downstream motor circuits (Fotowat and Engert 2023). While our explorations did not segregate the dimming and moving edge components of the loom stimulus, we see similar responses that align with the dim‐sensitive responses reported by Fotowat and Engert. It would be interesting in the future to investigate whether isoluminant checkerboard looms (mimicking the expanding edges of the loom) transfer any information between tecta pertaining to the location of the stimulus in the visual space, where there may be intersections between the circuits that detect the expanding edges and those that identify the location of potential looming threats. Furthermore, reports of stimulus‐specific adaptation show that a novel stimulus introduced within a sequence elicits an increase across both behavior and neural responses and suggest that distinguishable neural pathways process the different stimuli (Netser et al. 2011; Vinken et al. 2017; Wilde et al. 2025).

The results from our fixed, bilateral, and anterior–posterior paradigms, therefore, suggest that neural adaptation is likely mediated by local circuitry. In this context, “local” describes elements of the visual and sensorimotor network that are specific to a region in visual space, not necessarily meaning that the key circuits are tightly restricted spatially in the brain. That said, there are multiple lines of evidence from our results supporting the existing idea that local circuits in the tectum are making key contributions to this targeted neural adaptation (Dutta and Gutfreund 2014; Fernandes et al. 2021; Fotowat and Engert 2023; Marquez‐Legorreta et al. 2022; Mysore et al. 2010; Suzuki et al. 2019; Temizer et al. 2015). First, these visual loom stimuli are robustly represented in the tectum (Figure 3A), stimulating a higher proportion of tectal neurons than in any other brain region that we investigated. Like most of the brain regions that we analyzed, the tectum has strong laterality of responses (Figure 3D), explaining why the alternate looms in the bilateral stimulus train could be processed separately and show a significantly strong adaptation to looms restricted to one position of the visual space, suggesting a critical role in the underlying processing (Figure 5A). Finally, the tectum was the only region analyzed in which there was a significant spatial separation between neurons responding to the reference and alternate looms in the anterior–posterior stimulus train. This provides a mechanism whereby the tectum could distinguish stimuli coming from different parts of the same eye field, and this distinction is a requirement for the stimulus‐specific brain‐wide adaptation that we report. Other brain regions' responses followed similar trends to tectal activity, wherein they adapted much more rapidly in the “fixed position” train relative to other stimulus trains. These regions include the tegmentum, pretectum, and thalamus, which have all previously been implicated in varying visuomotor transformations, where these regions detect and process stimulus information such as luminance and motion, which are crucial for generating appropriate behavioral responses (Heap et al. 2018; Helmbrecht et al. 2018; K. Wang et al. 2020). This suggests that within our paradigm, these regions may be involved in the sensory transformations that process and relay information such as stimulus location to downstream structures that drive adaptation.

This reliance on repeated stimulation of the same part of the visual field may also explain a discrepancy that has been observed between visual habituation in free‐swimming versus head‐immobilized larval zebrafish. Past studies have found that immobilized zebrafish adapt to repeated looms more quickly than larvae that receive looms while swimming freely (Fotowat and Engert 2023; Marquez‐Legorreta et al. 2022). In the former case, the repeated loom is delivered to the same retinal position, since the animal is stationary, whereas in the latter case, the animal's movements mean that different retinal positions will be targeted each time. Another explanation is simply that the futility of attempting to swim in a tethered preparation causes the larvae to “give up,” thus eliminating behavioral responses in the head‐embedded preparation versus free swimming (Mu et al. 2019).

In a free‐swimming animal, this may be an adaptive solution. Given the dire consequences of ignoring predatory threats, habituation to looming stimuli should occur conservatively. The historical context of an animal's experience may modulate the saliency of the stimulus it faces, where a stimulus that differs from a previous stimulus may be perceived as more salient and subsequently influence how the animal habituates. In a setting where there is predation, including frequent predatory attacks, it is important that the prospective prey remain vigilant and responsive. Assuming that repeated predatory strikes would come from different positions in visual space, the arrangement that we describe would lead to modest habituation to real predators. Instead, it might more likely take effect when an innocuous stimulus happens repeatedly in a particular position in space.

A limitation of our current study is a lack of behavioral information about the impacts of off‐target stimuli on habituation. Movements of the animals in our head‐embedded preparation are infrequent and inconsistent, making them a poor proxy for free‐swimming habituation. In future experiments, implementing close loop stimulation in our free‐swimming experimental setup can help bridge this gap between behavioral habituation and neural adaptation. Furthermore, it will be interesting to explore the degree to which this approach toward habituation is maintained in other animals with a variety of different ethological contexts or internal states, and whether there are clear relationships between animals' habituation strategies and their surroundings and selective pressures. It will also be interesting to characterize the circuits in the tectum and/or other brain regions mediating their spatially restricted habituation.

## 4 Materials and Methods

### 4.1 Animals

All experiments were conducted in accordance with the University of Queensland Animal Welfare Unit's ethics approval number SBS/341/19. Larval zebrafish of the tüpel longfin nacre (TLN) strain were used at 6 dpf. Larvae were raised in E3 media (distilled water with 10% Hanks solution, consisting of 137 mM NaCl, 5.4 mM KCl, 0.25 mM Na_2_HPO_4_, 0.44 mM KH_2_PO_4_, 1.3 mM CaCl_2_, 1.0 mM 654 MgSO_4_, and 4.2 mM NaHCO_3_ at pH 7.2) with methylene blue in 10 cm diameter Petri dishes at a density of 50 larvae per dish. TLN fish expressed the transgene *elavl3:H2B‐GCaMP6s* (ZFIN identifier: ZDB‐TGCONSTRCT‐141023‐1, RRID: Addgene_59530) for pan‐neuronal nuclear expression of calcium indicator GCaMP6s (Freeman et al. 2014)

### 4.2 Calcium Imaging Experiments

**FIGURE 6:**
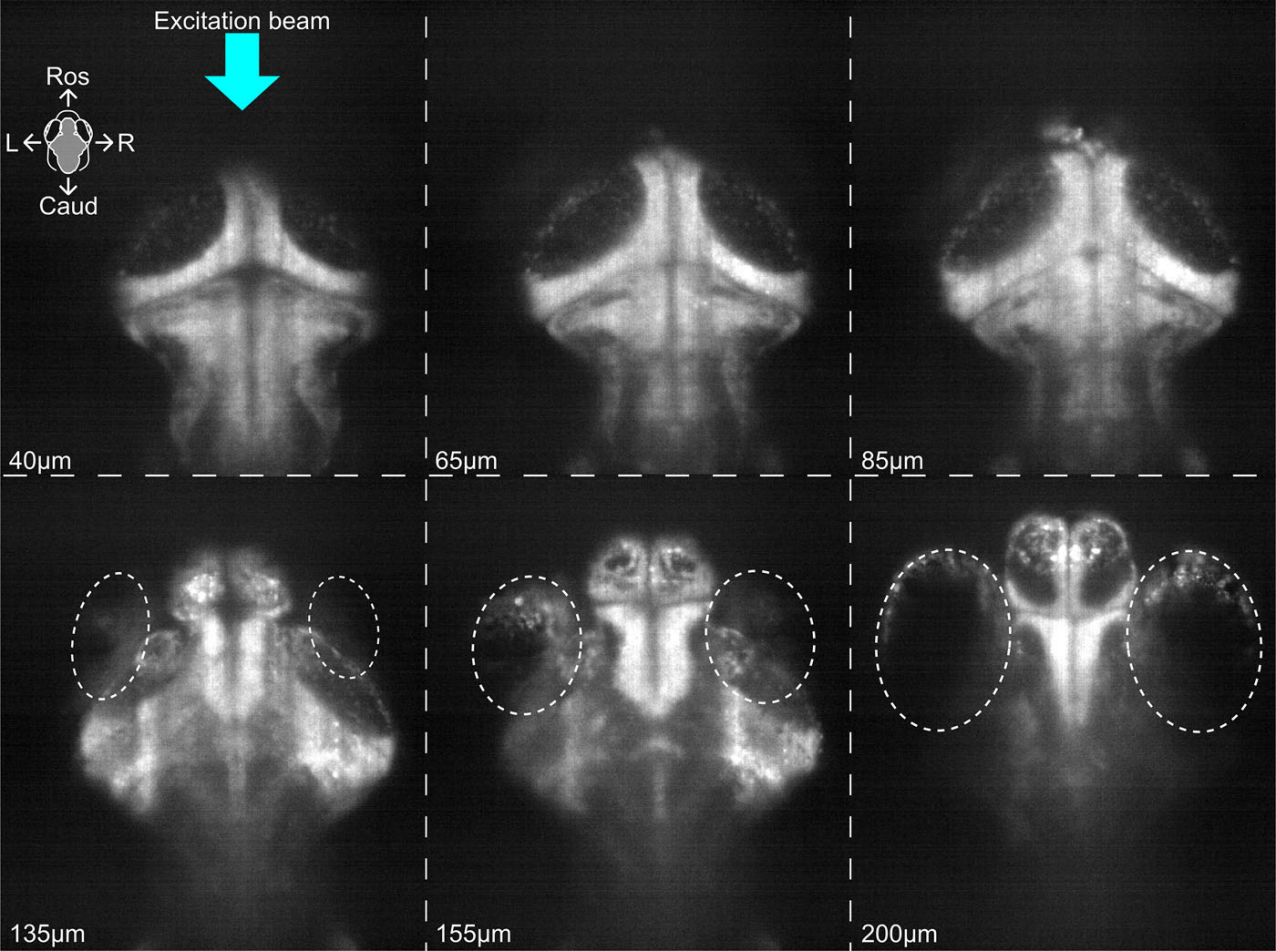
Raw data showing *z*‐slices through an exemplar brain from the light‐sheet calcium imaging set up. The excitation beam comes from the front of the animal, as shown by the arrow. The depth below the dorsal surface is labeled for each slice, and the approximate positions of the eyes are indicated (dashed ovals) in slices at depths of 135, 155, and 200 µm.

Larvae were mounted in 2% low melting point agarose (Sigma: A9045, RRID:SCR_008988.) in a custom cubic 3D‐printed chamber (24 mm width, 24 mm length, 20 mm height), with glass coverslips forming the four walls (Figure 1A). The chamber was filled with E3 media (without methylene blue) after agarose was set, as previously described (A. W. Thompson and Scott 2016). Larvae were then imaged using a custom‐built light‐sheet microscope (Taylor et al. 2018; Vanwalleghem et al. 2020). To avoid the strong flickering visual stimuli that result from side illumination, we presented the excitation light sheet from the front of the animal only (Figure 1A). This carries with it the risk that brain regions caudal to the eyes (which have an opaque retinal pigmented epithelium) will not be illuminated and that we will therefore fail to detect responses in those regions. Fortunately, we find that the use of a diffusive light sheet (Taylor et al. 2018) covers these regions reasonably well and that we see responses in brain regions at risk of masking by the eyes (Figure 6). However, we still acknowledge the potential limitation of underrepresenting responses from these regions caudal to the eyes when interpreting results. A total of 50 *z*‐planes over a range of 250 µm dorso‐ventrally were imaged to capture data from the full volume of the brain at a rate of 2 Hz. Images were binned 4× to a final resolution of 640 × 540 pixels in tagged image file format (TIFF), as described (Constantin et al. 2020; Favre‐Bulle et al. 2025; Wilde et al. 2024).

### 4.3 Visual Loom Stimulation

Stimulus trains were made up of a series of repetitive looms. Looms started as a black disc on a gray background that grew hyperbolically to a diameter of 33.7 mm over the course of 5 s, followed by a 15‐s period of a linear fade to gray (Figure 1B). Interstimulus intervals between each loom ranged from 25 to 35 s, randomly scattered throughout the stimulus train. A recovery period of 5 min was included in the stimulus train, after which one last loom was presented (Figure 1B). Within each train, some of the looms (referred to as reference looms) were delivered in the right anterior eye field, while the other looms (alternate looms) were delivered to different locations in visual space. The first train, fixed position, had alternate looms in the front right field, matching the reference looms (*n* = 8). In the anterior–posterior train, alternate looms were delivered in the posterior right eye field (*n* = 9). The bilateral train had alternate looms in the anterior left eye field (*n* = 8). Finally, the absent train served as a negative control, with no loom occurring during alternate trials (*n* = 10) (Figure 1C). The temporal properties and order of reference and alternate looms were kept consistent across the different stimulus trains so that reference and alternate responses could be compared across groups (Figure 1B). Looms were presented to fish on 75 × 55 mm LCD generic PnP monitors (800 × 600 pixels) covered with a 550 nm cut‐on wavelength filter and were positioned on both sides (left and right) 37.5 mm from the center of the chamber (Figure 1B).

### 4.4 Extraction of Fluorescent Traces of Calcium Activity

Calcium imaging data were reformatted into separate TIFF files for individual *z*‐planes (50 files per fish) in ImageJ v1.52c (RRID:SCR_003070). Motion correction was performed using the Non‐Rigid Motion Correction (NoRMCorre) algorithm, and fluorescence traces were extracted and demixed from the time series using the CaImAn package (version 0.9, 1) (RRID:SCR_021152) (Giovannucci et al. 2019; Pnevmatikakis et al. 2016). After the fluorescence traces were extracted, filtered noise was added back to each ROI to account for possible negative signals (Vanwalleghem et al. 2021).

### 4.5 Registration to Reference Atlas for Anatomical Classification

We used Advanced Normalization Tools (ANTs) (RRID:SCR_004757) to register the ROIs to the H2B‐RFP reference of Zbrain (Avants et al. 2008, 2011; Randlett et al. 2015). Images from the SPIM experiments were warped to a common template previously acquired and registered to the ZBrain atlas (Favre‐Bulle et al. 2018; Randlett et al. 2015). The resulting warping parameters were applied to the xyz coordinates of the centroids of the ROIs to map them into the 294 brain regions defined in the Zbrain atlas, as previously described (Poulsen et al. 2021).

### 4.6 Analysis of Calcium Imaging Data

Analysis was conducted using MATLAB R2022b (RRID:SCR_001622) and GraphPad Prism v10.4.1 (RRID:SCR_002798). The first step in this analysis was to identify loom‐specific visual responses across the brain. To do this, three stimulus regressors were designed by placing a simulated calcium spike at the time points of the first four looms (Poulsen et al. 2021). These regressors were “both”: placing spikes where either alternate or reference looms appeared, “reference preferring”: placing a spike only when a reference loom appeared, and finally, “alternate preferring”: placing a spike only when an alternate loom appeared (Figure 2, top). Next, to remove responses from any behavioral movement, we built motor regressors for each fish based on the motion correction performed during preprocessing and correlated these to all ROIs during the times when no stimuli were presented.

**FIGURE 7:**
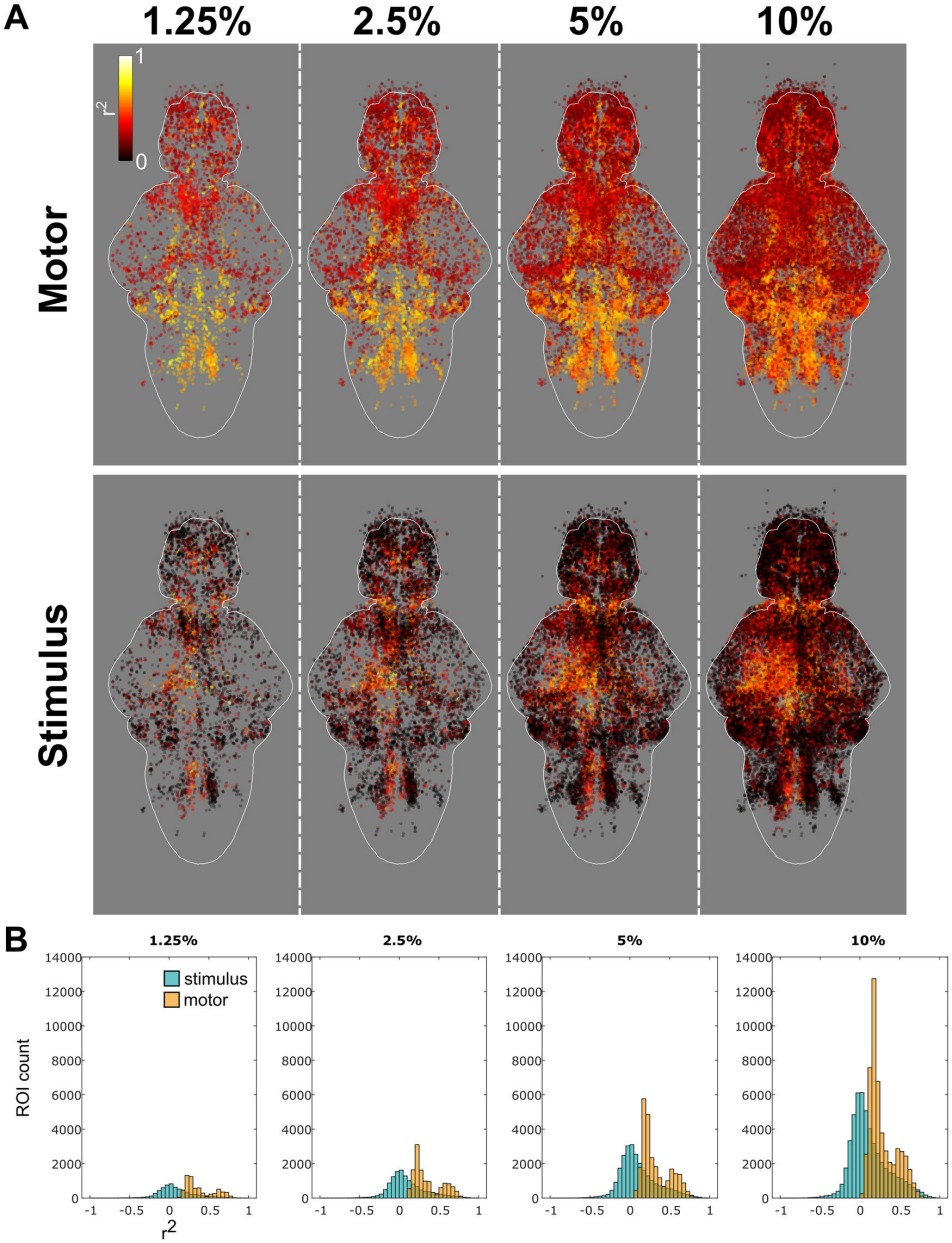
Exploring thresholds for the motor regressor. (A) The top row shows the distribution of ROIs, colored by their *r* ^2^ value at different thresholds (1.25%, 2.5%, 5% and 10%) to their motor regressors. The bottom row shows these same ROIs but now colored by their *r* ^2^ value to the stimulus regressor. (B) Histogram distribution of the *r* ^2^ values of both the motor and stimulus regressors at each threshold.

**FIGURE 8:**
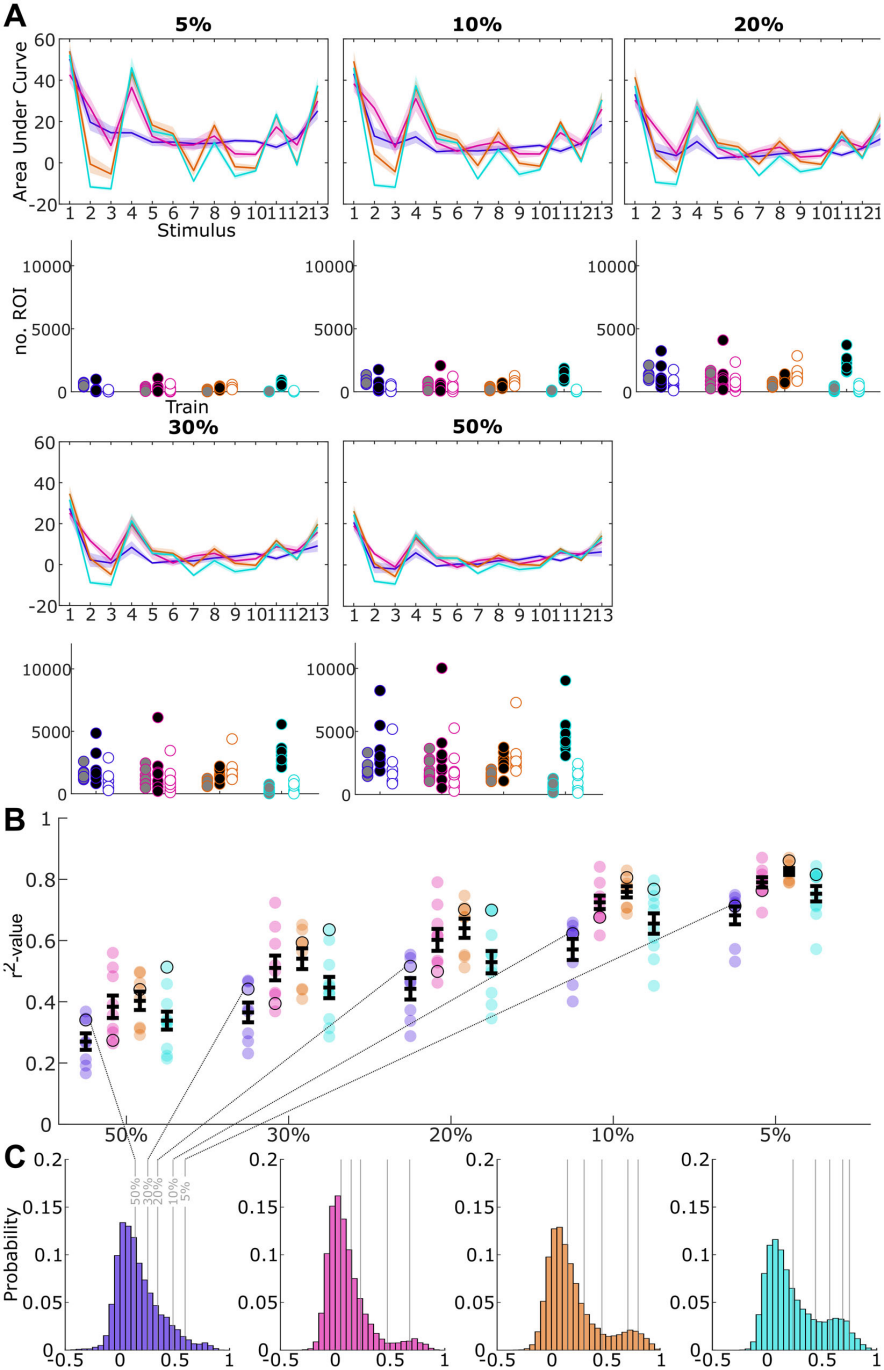
Exploring different thresholds for stimulus regressors. (A) Top is the habituation of reference responsive ROIs across the different stimulus trains, and bottom shows the number of ROIs in each of the visually responsive categories (gray = both, black = ref only, white = alt only) at different thresholds of 5%, 10%, 20%, 30%, and 50%. (B) The average *r* ^2^ value of ROIs surpassing the threshold for each fish in each stimulus train. (C) Histograms of *r* ^2^ values in example fish from each of the stimulus trains; gray lines represent where the threshold was placed for that fish.

The top 20% of ROIs with the highest *R* ^2^ value toward each stimulus regressor per fish were considered to be visually responsive, and the top 2.5% of ROIs with the highest *R* ^2^ value toward their respective motor regressor were excluded from the visual responses. Analysis of other thresholds for both the stimulus and motor regressor shows that the findings we report are not unique to 20% or 2.5% and were chosen as they strike a balance between filtering out motor activity and maintaining interesting sensory information (Figures 7 and 8). We calculated the proportion of visually responsive ROIs per brain region based on the total number of segmented ROIs (Figure 3A). Then, we calculated the average proportion of these ROIs that fell into the “both,” “reference preferring,” or “alternate preferring” categories (Figure 3B).

We calculated laterality and polarity indices for the bilateral and anterior–posterior trains, respectively, based on their “reference preferring” and “alternate preferring” responses (Figure 3C,D). To calculate the laterality index, each brain region was split into a right and left hemisphere, and the sum of “reference preferring” ROIs in the left hemisphere was subtracted from the sum of “reference preferring” ROIs in the right hemisphere. This was then normalized to the total sum of “reference preferring” ROIs within that entire brain region. The same calculation was then performed for the “alternate preferring” ROIs and the “both” ROIs. Significance for the paired data points between “reference preferring” and “alternate preferring” was tested using the Mann‒Whitney *U* test to identify whether position‐specific responses across the left and right visual fields evoked a similar distribution of responses between the two brain hemispheres. To calculate the polarity, the central point of each brain region's visual responses was determined by finding the mean coordinates of all visually responsive ROIs. Next, the relative 2D angle (incorporating rostral‐caudal and medial‐lateral axes) of all “reference preferring” ROIs was calculated from this central point and averaged across fish. The same calculation was then performed for the “alternate preferring” ROIs and the “both” ROIs, yielding the relative angle of these responsive ROIs to the centroid of the brain region. Significance for the paired data points between “reference preferring” and “alternate preferring” was tested using the Mann‒Whitney *U* test to find whether position‐specific responses were localized to distinct spatial distributions within each brain region.

To look at adaptation across the entire brain, we pooled “reference preferring” and “both” ROIs as “reference responsive.” By selecting these responses, we could address how alternate stimuli influenced adaptation to reference stimuli, as reference stimuli were kept constant across the four different trains and served as comparison points for analyses. To investigate the extent of adaptation, we calculated the adaptation index of each ROI at all reference loom time points. First, we calculated the area under the curve for calcium responses at each stimulus presentation to yield one data point per loom. Loom 4 was the second reference loom to appear in the stimulus sequence (Figure 4B) and therefore provided the clearest readout of adaptation for each of our four stimulus trains. Adaptation indices were then calculated by subtracting the response at Loom 4 from the response at Loom 1 and then normalizing this value to Loom 1. The result gives a readout of how much an ROI was adapted by the fourth stimulus, with a value of 1 representing complete adaptation, 0 representing no adaptation, and negative values representing an increase in response in the fourth trial relative to the first (Figure 4C). To compare overall brain‐wide adaptation between the four trains, the median adaptation index was calculated for each fish, and significance was tested using the Kruskal‒Wallis test and corrected for multiple comparisons with Bonferroni correction (Figure 4D,E). A pipeline of steps taken during this analysis can be found in Figure 1D.

Finally, to look at adaptation within the different visually responsive brain regions, we similarly looked at the adaptation index distribution of “reference responsive” ROIs across the stimulus trains. To compare the regional adaptation between the four trains, the median adaptation index was calculated for each fish, and significance was tested using the Kruskal‒Wallis test and corrected for multiple comparisons with Bonferroni correction (Figure 5A).

## Author Contributions

T.M., E.M.L., M.P., and E.K.S. contributed to the experimental design of this project. I.F.B. built and maintained the calcium imaging microscope used for data collection. G.V. contributed to the processing of calcium imaging data. E.M.L., M.P., M.W., and E.S. helped guide data analysis. T.M. and E.M.L. collected and analyzed the data. E.K.S. and T.M. contributed to the writing of the manuscript. All authors contributed to the production of the manuscript and approved the final version.

## Data Availability

All data relevant to this manuscript can be found at https://doi.org/10.26188/32720853.
